# Temporal changes in allergen-specific IgE sensitization patterns in Xiamen, 2019–2023: a retrospective cross-sectional analysis

**DOI:** 10.3389/fpubh.2026.1916258

**Published:** 2026-09-11

**Authors:** Linlin Qu, Xiujuan Li, Ruopei Wang, Kai Zhang, Jinting Geng, Meiying Su, Lanlan Lian, Chunhua Yang, Houzhao Wang, Xiaojun Jing

**Affiliations:** 1 Department of Clinical Laboratory, Xiang’an Hospital of Xiamen University, School of Medicine, Xiamen University, Xiamen, Fujian, China; 2 Department of Clinical Laboratory, Women and Children’s Hospital, School of Medicine, Xiamen University, Xiamen, Fujian, China; 3 School of Medicine, Xiamen University, Xiamen, Fujian, China; 4 Department of Infectious Diseases and Hepatology, Xiang’an Hospital of Xiamen University, School of Medicine, Xiamen University, Xiamen, Fujian, China; 5 Department of Equipment and Materials, Xiang’an Hospital of Xiamen University, School of Medicine, Xiamen University, Xiamen, Fujian, China

**Keywords:** allergen, allergy, COVID-19 pandemic, epidemiological characteristics, immunoglobulin E, retrospective analysis, Xiamen

## Abstract

**Background:**

The COVID-19 pandemic has induced substantial changes in environment, lifestyle, and health behaviors. We observed temporal associations between the pandemic period and declining sIgE positivity rates; however, causality cannot be established due to the retrospective observational design. Distinguishing between asymptomatic sensitization (positive specific IgE without clinical symptoms) and true allergic disease remains critical. This study aims to describe the trends in allergen-specific IgE (sIgE) positivity—as a marker of sensitization—in the Xiamen population from 2019 to 2023, a surrogate marker of sensitization that does not equate to clinical allergy, providing insights into population-level immunological changes during the pandemic.

**Methods:**

We performed a retrospective cross-sectional analysis of 3,207 serum sIgE testing records (3,210 initial records minus 3 exclusions) from patients who underwent allergen screening at our hospital between January 2019 and December 2023. These records include repeated tests from some individuals; a subset of 2,784 records from 2,750 unique patients was used for GEE modeling to account for within-individual correlation. Data on sensitization to inhalant allergens (e.g., dust mites, cat dander, cockroach) and food allergens (e.g., egg white, crab, milk) were analyzed. Age and sex distributions were examined, though detailed information on clinical symptoms (e.g., eczema, rhinitis), comorbidities, and longitudinal follow-up of individual patients was not available.

**Results:**

Among 3,207 testing records, 1,454 (45.34%) were positive for sIgE to one or more allergens. The annual positivity rate declined sharply from 76.07% (89/117) in 2019 to 32.26% (519/1,609) in 2023 (*p* < 0.001). Dust mites were the most prevalent inhalant allergens, with about a third of all testing records showing sensitivity (35.30%, 1,035/3,207), followed by cockroach and cat hair. For food allergens, egg white, crab, and milk ranked highest. Males showed higher positivity rates for both inhalant and food allergens compared to females, and the difference was statistically significant (*p* < 0.01). Most allergens showed age-related differences, with the exception of cat hair, dog epithelium, crb and ragweed; cockroach showed statistically significant differences across age groups (*χ*^2^ = 32.89, *p* < 0.001). We observed temporal associations between the pandemic period and declining sIgE positivity rates; however, these findings should be interpreted with caution due to the observational study design and potential selection bias.

**Conclusion:**

This study documents a significant decline in sIgE positivity rates for both inhalant and food allergens in Xiamen from 2019 to 2023. To our knowledge, this is the first report from Xiamen spanning the full COVID-19 pandemic period, providing foundational data for future hypothesis-driven research. Sex- and age-specific differences in sensitization patterns were observed. However, based on the current dataset alone, it is not possible to determine whether these changes reflect true shifts in clinical allergic disease or result from alterations in testing practices, population composition, or other confounders. Future research must integrate detailed clinical phenotyping (including age-stratified symptom data, comorbidities such as eczema, and longitudinal follow-up) and lifestyle/environmental measurements to identify modifiable factors for preventing allergy development—not merely managing sensitization.

## 1 Introduction

Allergic diseases encompass a spectrum of conditions triggered by an abnormal immune response to allergens, encompassing allergic rhinitis, allergic asthma, allergic conjunctivitis, atopic dermatitis, and food allergies. The World Allergy Organization (WAO)’s White Paper reports that 30–40% of people worldwide either have or have had some form of allergic disease, and their prevalence continues to rise globally (1, 2). A pivotal factor in the development of these conditions is IgE-mediated type I hypersensitivity reactions (3). Identifying allergens is critical for adopting effective preventive and diagnostic measures, and one reliable method for achieving this is through *in vitro* testing of serum allergen-specific IgE (sIgE) (4). SIgE testing offers several advantages over skin tests, including more stable results, uninfluenced by drug-induced skin reactions, and a lower risk profile (5, 6). However, it also has notable disadvantages, such as higher cost, the requirement for venipuncture, limited detection panels that may miss relevant allergens, and results that do not always correlate with clinical symptoms due to cross-reactivity or asymptomatic sensitization. In contrast, *in vivo* allergy testing methods (e.g., skin prick tests) provide rapid, direct results but are limited by interference from antihistamines, the risk of systemic allergic reactions, unsuitability for patients with severe dermatitis or dermographism, and poor reproducibility. Therefore, both *in vitro* and *in vivo* tests have complementary roles and limitations, and the choice should be guided by the clinical context. In this study, all sIgE test results were retrospectively retrieved from electronic medical records of patients who visited the hospital for suspected allergic diseases between 2019 and 2023; no sera were prospectively collected specifically for this research. This testing method helps healthcare providers understand the etiology and sensitization status of patients with allergic diseases, guiding personalized treatment plans (7). However, epidemiological survey data on allergens must be updated regularly due to the influence of geography, environment, and climate (8). The COVID-19 pandemic has significantly impacted society, prompting strict non-pharmaceutical interventions (NPIs) such as wearing masks, restricting outdoor activities, maintaining social distancing, limiting gatherings, and spending prolonged periods indoors. These measures temporarily altered the types and levels of human exposure to environmental allergens. Consequently, studies have reported a decrease in infectious diseases during the pandemic but an increase in severe allergic diseases (9–11).

Researchers are starting to unpack the impact of COVID-19 on allergic diseases. In the US, a large retrospective cohort study drawing on an electronic health records database of more than 118 million patients analyzed three cohorts totaling approximately 6.05 million individuals and found that COVID-19 was associated with a higher risk of asthma (HR = 1.656) and allergic rhinitis (HR = 1.272) (12). In China, however, different trends have been observed. A longitudinal analysis in Shenzhen followed 43,314 children and noticed the timing of sensitization varied by allergen. Dust mite sensitization increased markedly after 2020, but cat dander sensitization did not peak until 2022 (13). Another study in Hangzhou focused on children with atopic dermatitis. Out of 8,797 kids, COVID-19 restrictions led to a dramatic drop in sensitization risk (OR = 0.40), but once restrictions eased, the risk returned to higher levels, especially for house dust mites (OR = 1.33) (14). These geographically heterogeneous findings highlight the need for region-specific epidemiological surveillance, particularly in understudied areas such as Xiamen.

To address this gap, our research had two key objectives: first, to describe the epidemiological characteristics of common inhalant and food allergens in Xiamen from 2019 to 2023; second, to evaluate the impact of the COVID-19 pandemic on allergen sensitization patterns, drawing on sIgE data from hospital records to provide updated epidemiological evidence for local allergy care.

## 2 Participants and methods

### 2.1 Study objects

Using data extracted from electronic medical records and routine clinical laboratory databases, this retrospective, cross-sectional study enrolled patients with suspected allergic diseases (including allergic rhinitis, bronchial asthma, atopic dermatitis, allergic conjunctivitis, and urticaria) who underwent serum allergen-specific IgE (sIgE) antibody screening at Xiang’an Hospital Affiliated to Xiamen University between January 2019 and December 2023.

A total of 3,210 testing records were retrospectively enrolled. After excluding 2 records with incomplete demographic or clinical data and 1 record with isolated CCD positivity (sIgE-CCD ≥ 0.35 kU/L with no concurrent non-CCD allergen positivity), 3,207 testing records (1,645 males, 1,562 females) were included in the descriptive analysis. These 3,207 records include repeated testing episodes from some patients over the study period. For the subsequent Generalized Estimating Equations (GEE) modeling, we further excluded 423 same-day duplicate records to avoid pseudo-replication, yielding 2,784 retained testing records. These records corresponded to 2,750 unique patients, as 34 records represented repeat visits from the same individuals on different days (mean 1.01 records per patient).

The baseline demographic and clinical characteristics of the study population, stratified by year, are summarized in Supplementary Table S1. Age was non-normally distributed and therefore presented as median with interquartile range, while categorical variables were expressed as frequencies and percentages.

### 2.2 Serum allergen sIgE testing and interpretation

Serum allergen-specific IgE (sIgE) testing was performed on routine clinical blood samples according to the hospital’s standard protocols. sIgE levels were measured using the EUROIMMUN blot kit (EUROIMMUN Medizinische Labordiagnostika AG, Lübeck, Germany), the standardized panel routinely used in our hospital’s clinical practice for suspected allergic disease workup, which quantifies 19 allergen-specific IgE antibodies. It covers the most clinically relevant inhalant and food allergens prevalent in southeastern China, including dust mites, cockroaches, common food allergens, and regional seafood allergens. Inhalant allergens comprised tree mix (willow, poplar, elm), ragweed, mugwort, dust mite mix (*Dermatophagoides pteronyssinus* and *Dermatophagoides farinae*), house dust, cat hair, dog dander, cockroach, mold mix (Penicillium, Cladosporium, Aspergillus, Alternaria), and *Humulus scandens*. Food allergens included egg white, milk, peanut, soybean, beef, mutton, marine fish mix (cod, lobster, scallop), shrimp, and crab. Results were scanned and interpreted using EUROLineScan software (EUROIMMUN), with negative and positive controls verified for each run. The same software version (EUROLineScan 3.2) was used consistently throughout the study period (2019–2023).

A sIgE level of ≥0.35 kU/L was deemed positive and categorized semi-quantitatively into six grades, from grade 1 (0.35 to <0.70 kU/L) to grade 6 (≥100.00 kU/L). CCD positivity was evaluated using the same kit. To ensure accuracy, patients were excluded from the final analysis if they met either of the following criteria: (1) CCD-only positivity, defined as sIgE-CCD ≥ 0.35 kU/L with no concurrent positivity to any non-CCD allergen; or (2) multiple unexplained positives, defined as positivity to ≥3 unrelated allergens without corresponding clinical manifestations.

### 2.3 Statistical analysis

Data analysis was conducted using SPSS 27.0 (IBM Corp., Armonk, NY, USA). Categorical variables were summarized as frequencies and percentages. Group comparisons of sIgE positivity rates were performed using the chi-square test, or Fisher’s exact test when expected cell counts were <5. For overall comparisons across years or groups, unadjusted chi-square tests were used; for post-hoc pairwise comparisons, Bonferroni correction was applied with a corrected significance level of *α*′ = 0.005 for 10 comparisons (0.05/10). A two-tailed *p* < 0.05 was considered statistically significant for overall comparisons.

To account for within-individual correlation due to repeated tests from the same patient, Generalized Estimating Equations (GEE) with a binary logistic link function were applied in the multivariable analysis. The model included 2,784 testing records clustered by patient ID (2,750 unique patients, mean 1.01 records per patient; range 1–3) using an exchangeable correlation structure and robust standard errors (Huber-White sandwich estimator), with year (categorical, with 2019 as reference), age group (<3, 3–5, >5 to 18, >18 years, with <3 as reference), and sex (female as reference) as independent covariates, and overall sensitization (any sIgE positive) as the dependent variable. Adjusted odds ratios (aOR) and 95% confidence intervals (CI) were calculated. A test for linear trend across years was performed by treating year as a continuous variable in the model.

## 3 Results

### 3.1 General information

A total of 3,207 testing records from patients with suspected allergic diseases were analyzed for descriptive statistics between January 2019 and December 2023. All enrolled patients underwent serum sIgE testing for the same panel of 19 allergens (inhalant and food, as detailed in Methods). The overall positive rate of allergen-specific IgE (sIgE) over these years was 45.34% (1,454/3,207). As shown in Table 1, the annual positive rates were 76.07% in 2019, 68.86% in 2020, 63.66% in 2021, 50.30% in 2022, and 32.26% in 2023, with a significant overall difference across years (*χ*^2^ = 275.99, *p* < 0.001).

**Table 1:** Annual comparison of serum allergen-specific IgE positivity rates based on testing records from patients with suspected allergic diseases in Xiamen, 2019–2023.

Year	Positive/total	Positive rate	*χ* ^2^	*p*
2019	89/117	76.07%		
2020	188/273	68.86%		
2021	240/377	63.66%		
2022	418/831	50.30%		
2023	519/1,609	32.26%		
All	1,454/3,207	45.34%	275.99	<0.001

Pairwise comparisons with Bonferroni correction for 10 comparisons (adjusted significance level *α*′ = 0.005) are presented in Supplementary Table S2. Compared to 2019, the annual positivity rates in 2022 and 2023 were significantly lower (adjusted *p* < 0.001), whereas the differences between 2019 and 2020 (adjusted *p* = 1.000) and between 2019 and 2021 (adjusted *p* = 0.130) were not statistically significant. Numerically, the rate continued to decline from 2022 to 2023, reaching the lowest point in 2023.

**Figure 1:**
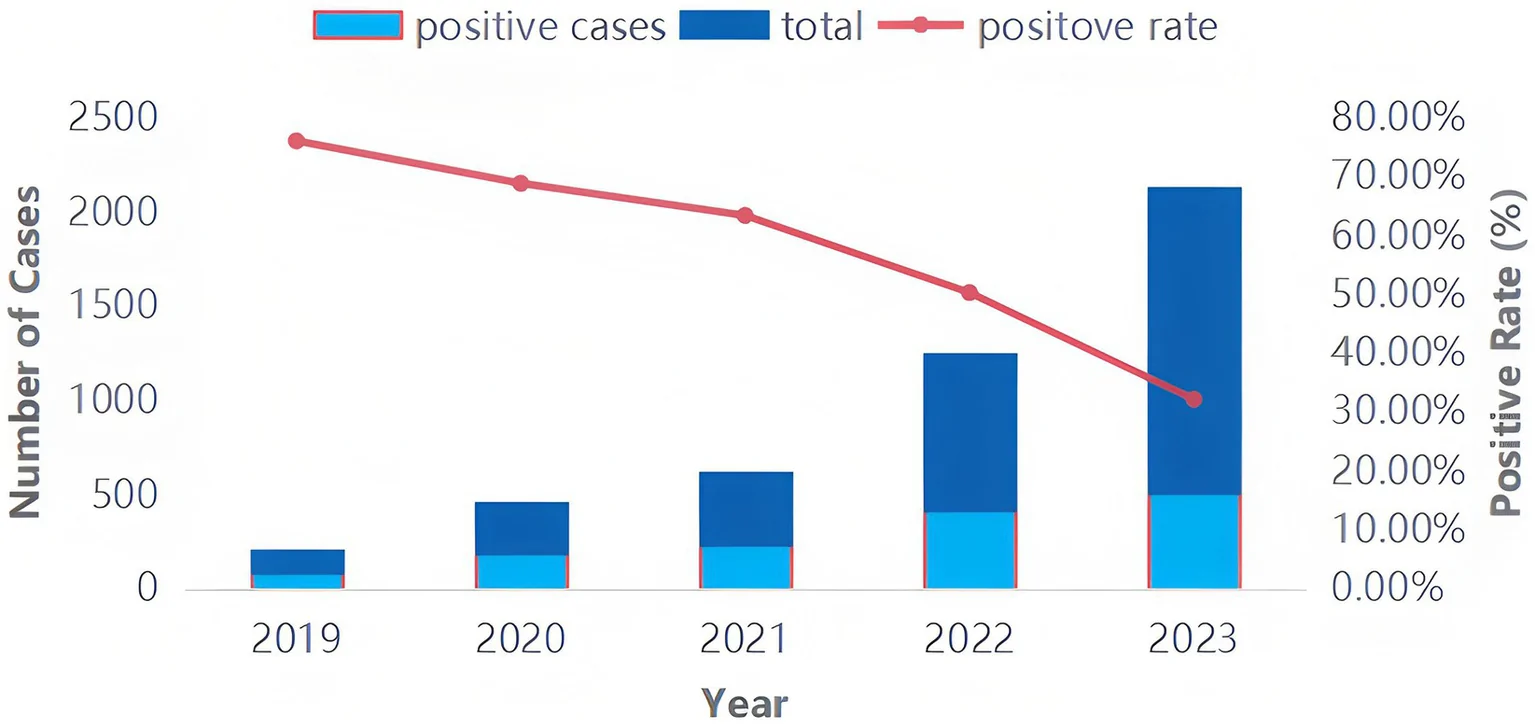
Annual total testing cases, sIgE positive cases, and positive rate of allergen-specific immunoglobulin E (sIgE) testing from 2019 to 2023. Light blue stacked bars represent positive sIgE cases; dark blue stacked bars indicate the total number of tested records each year. The red line with circular markers shows the yearly sIgE positive rate (right vertical axis). Error bars denote measurement variability/standard deviation.

Supplementary Table S1 further illustrates the changing composition of the study population across years. The median age decreased from 32 years (IQR: 4–32) in 2019 to 11 years (IQR: 4–34) in 2021, then rebounded to 22 years (IQR: 6–36) by 2023. The proportion of inpatient visits increased from 0% in 2019 to 19.9% in 2023, reflecting the expanding scope of testing from a primarily outpatient referral population to a broader hospital-based cohort. Given these significant demographic shifts, particularly in age and inpatient proportion, we further performed Generalized Estimating Equations (GEE) adjusting for these factors to assess the independent effect of study year on sensitization, as detailed in Table 2. These findings are summarized in Figure 1 and Table 1.

**Table 2:** Multivariable analysis for overall sensitization (any sIgE positive) using Generalized Estimating Equations (GEE).

Year	aOR^*^	95% CI	*p*
2019	Reference		
2020	0.68	0.41–1.12	0.132
2021	0.49	0.30–0.80	0.004
2022	0.3	0.19–0.48	<0.001
2023	0.24	0.15–0.37	<0.001

aOR, adjusted odds ratio; CI, confidence interval. Adjusted for age group (<3, 3–5, >5 to 18, >18 years) and gender (female as reference) using Generalized Estimating Equations (GEE) with exchangeable correlation structure and robust standard errors. Year was entered as a categorical variable with 2019 as the reference; test for linear trend across years was performed by treating year as a continuous variable (*p* for trend <0.001). * indicates *p* < 0.01.

The denominator for each year represents the number of testing records, not unique patients. Some patients contributed multiple records across different years.

### 3.2 Positive rate of each allergen from 2019 to 2023

**Figure 2:**
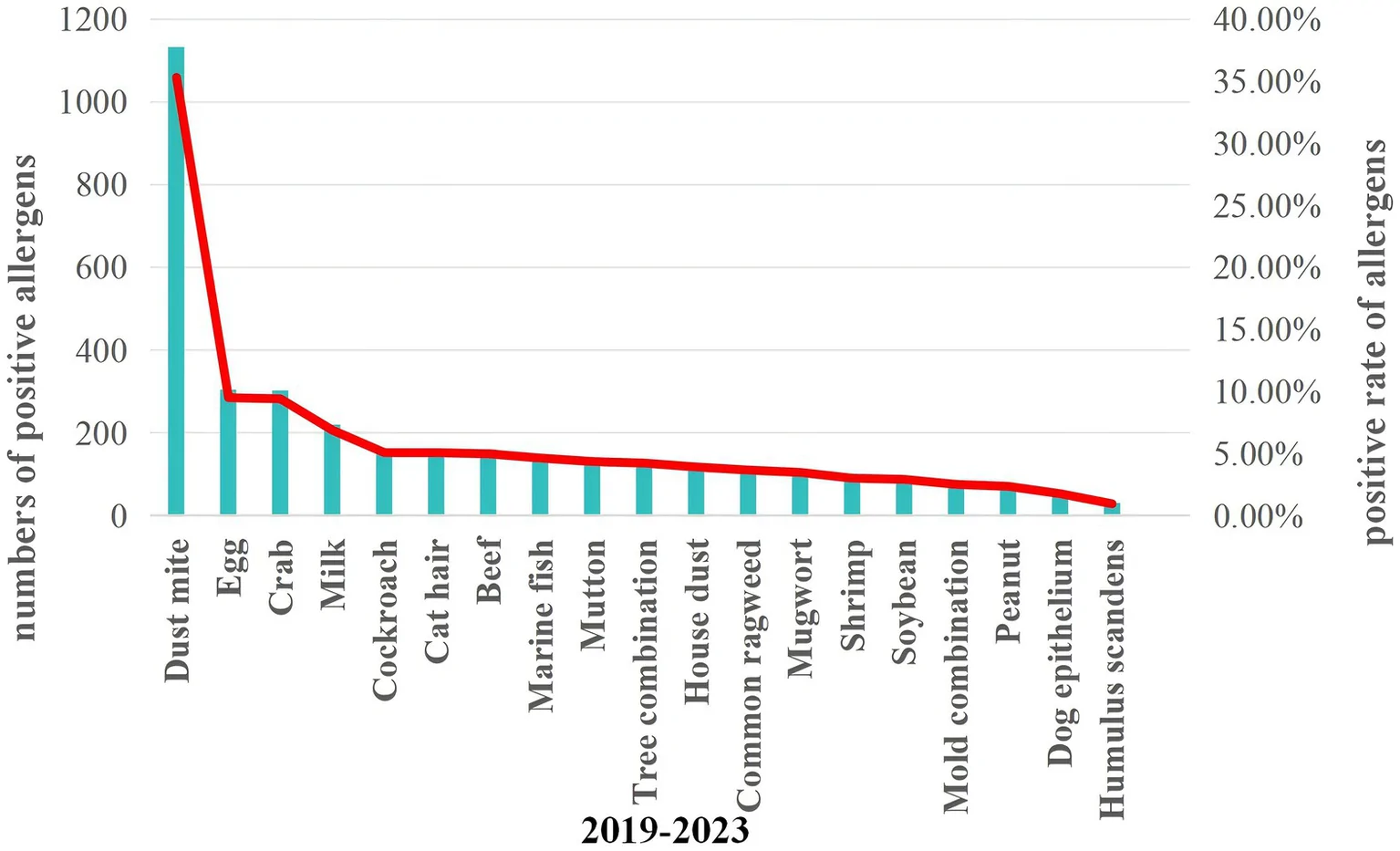
Absolute positive counts and positive rates of individual allergen-specific IgE (sIgE) among patients with suspected allergic diseases during 2019–2023. Light green bars represent the total number of positive detections for each allergen (left vertical axis: number of positive allergens). The red line with circular markers indicates the positive rate of each allergen (right vertical axis: positive rate of allergens). Allergens are ranked on the *x*-axis in descending order of absolute positive cases.

Dust mite showed the highest positivity rate (35.30%) among allergens from 2019 to 2023, followed by egg (9.51%), crab (9.42%), and milk (6.89%). Figure 2 illustrates the specific positivity rates of the 19 different allergens during this period.

### 3.3 Analysis of trends in allergen positive rates across different years

**Figure 3:**
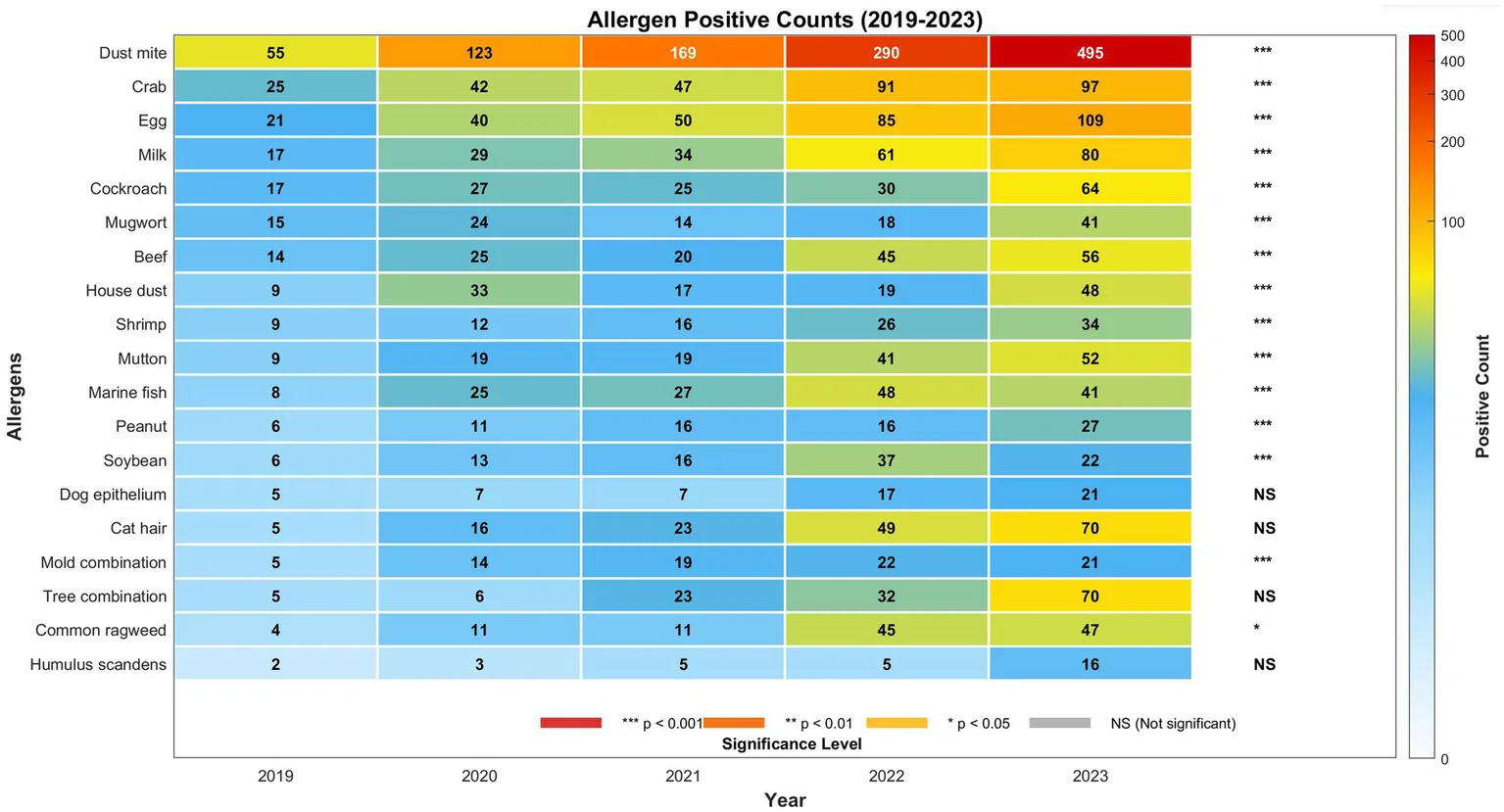
Heatmap showing annual positive counts of individual allergens detected by allergen-specific IgE testing from 2019 to 2023. Rows represent distinct allergens, while columns correspond to calendar years. The color gradient intensity indicates the magnitude of positive counts, with the right color bar denoting numerical values. The “Sig.” column on the far right summarizes interannual statistical significance: ^***^p < 0.001, ^**^p < 0.01, ^*^p < 0.05, and “NS” for non-significant differences.

The study conducted from 2019 to 2023 revealed a decrease in positive rates of inhaled allergens (excluding house dust) during the pandemic period, with significant differences compared to 2019. This trend was also observed for food allergens, showing a decline year by year. Statistical analysis (*p* < 0.05) supported these findings, as depicted in Figure 3 and Table 3. Multivariable logistic regression using GEE, adjusting for age group and sex, confirmed that year remained significantly associated with overall sensitization (Wald *χ*^2^ = 99.506, *p* < 0.001). Compared with 2019, the adjusted odds of sensitization were significantly lower in 2021 (aOR = 0.49, 95% CI: 0.30–0.80, *p* = 0.004), 2022 (aOR = 0.30, 95% CI: 0.19–0.48, *p* < 0.001), and 2023 (aOR = 0.24, 95% CI: 0.15–0.37, *p* < 0.001), whereas the reduction in 2020 did not reach statistical significance (aOR = 0.68, 95% CI: 0.41–1.12, *p* = 0.132; Table 2; detailed regression results in Supplementary Table S3). A significant downward linear trend was observed across the study period (*p* for trend <0.001). Despite the declining adjusted odds, the absolute number of positive records increased substantially for most allergens over the same period (dust mite: 55 → 495; crab: 25 → 97; egg: 21 → 109; milk:17–18).

**Table 3:** Allergen positive counts and rates of individual allergens based on testing records, 2019–2023.

Allergens	2019 (117)	2020 (273)	2021 (377)	2022 (831)	2023 (1,609)	*χ* ^2^	*p*
Dust mite	55	123	169	290	495	47.934	<0.01
Crab	25	42	47	91	97	59.045	<0.01
Egg	21	40	50	85	109	38.728	<0.01
Milk	17	29	34	61	80	28.721	<0.01
Cockroach	17	27	25	30	64	44.406	<0.01
Mugwort	15	24	14	18	41	61.597	<0.01
Beef	14	25	20	45	56	30.145	<0.01
House dust	9	33	17	19	48	62.262	<0.01
Shrimp	9	12	16	26	34	16.94	<0.01
Mutton	9	19	19	41	52	13.509	<0.01
Marine fish	8	25	27	48	41	37.576	<0.01
Peanut	6	11	16	16	27	16.858	<0.01
Soybean	6	13	16	37	22	28.075	<0.01
Dog epithelium	5	7	7	17	21	7.644	0.091
Cat hair	5	16	23	49	70	4.24	0.374
Mold combination	5	14	19	22	21	28.427	<0.01
Tree combination	5	6	23	32	70	6.377	0.173
Common ragweed	4	11	11	45	47	10.409	0.034
*Humulus scandens*	2	3	5	5	16	3.171	0.493

Data are presented as positive counts (*n*) with denominators representing the total number of testing records for each year (117, 273, 377, 831, and 1,609, respectively). Denominators are testing records, not unique patients. *χ*^2^ and *p* values are from chi-square tests for interannual comparisons across the 5 years.

### 3.4 Secondary analysis: differences in positive rates of inhalant and food allergens across different age groups

**Figure 4:**
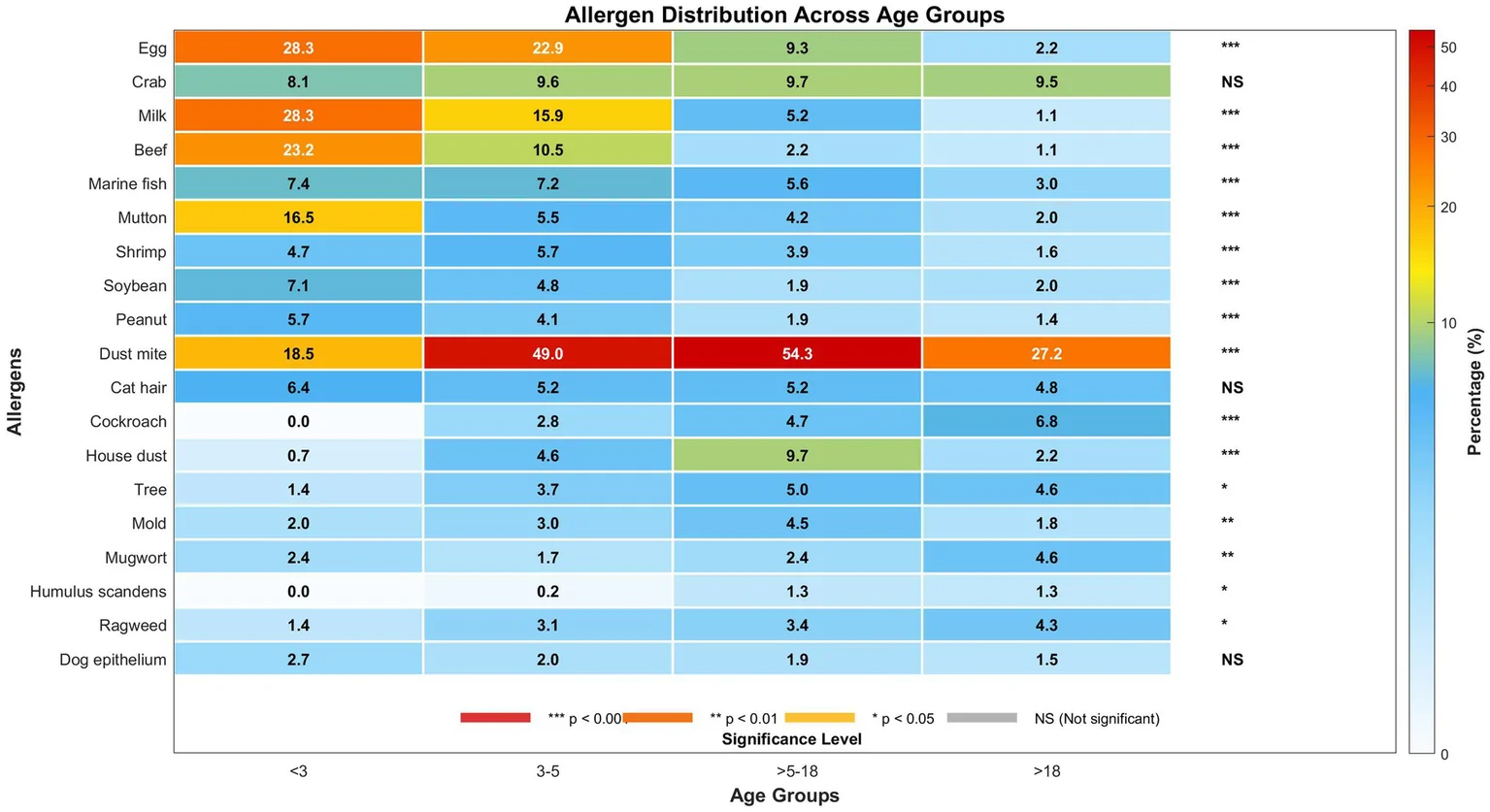
Heatmap displaying allergen-specific IgE (sIgE) positive rates (%) stratified by four age groups: <3 years, 3–5 years, >5 to 18 years, and >18 years. Rows represent individual allergens, columns correspond to age subgroups. Color gradient on the right bar quantifies positive percentage values, with deeper red indicating higher positivity. The “Sig.” column on the far right summarizes interannual statistical significance: ^***^p < 0.001, ^**^p < 0.01, ^*^p < 0.05, and “NS” for non-significant differences.

Although not the primary aim, we also examined age- and sex-related differences. As shown in Figure 4 and Table 4, the frequently detected inhalant allergens across all ages were dust mites, cat hair, tree pollen, and mold; the frequently detected food allergens were hen’s egg white, milk, beef, and mutton. However, the ranking varied by age. In infants (0–3 years), the top inhalant allergens included dust mites, cat hair, dog epithelium, and mugwort, while the top food allergens were hen’s egg white, milk, beef, and mutton. In preschool children (3–5 years), dust mites were the leading inhalant allergen (265/541, 48.98%), followed by cat hair (28/541, 5.18%), house dust (25/541, 4.62%), tree pollen (20/541, 3.70%), and mold (16/541, 2.96%). In school-age children (>5 to 18 years), dust mites, house dust, cat hair, and tree pollen dominated among inhalant allergens, and crab (60/621, 9.66%), hen’s egg white (58/621, 9.34%), seafood mix (35/621, 5.64%), and milk (32/621, 5.15%) among food allergens. In adults (≥18 years), the predominant inhalant allergens were dust mites, cockroach, cat hair, and tree pollen, while the top food allergens were crab, seafood mix, hen’s egg, and mutton. Significant differences in sIgE positivity rates across age groups were observed for most allergens, with the exception of cat hair, dog epithelium, and crab (*χ*^2^ = 0.702, *p* = 0.873); cockroach showed statistically significant differences across age groups (*χ*^2^ = 32.89, *p* < 0.001).

**Table 4:** Allergen positive rates across different age groups based on testing records (*n*, %).

Allergens	<3 (*n* = 297)	3–5 (*n* = 541)	>5 to 18 (*n* = 621)	>18 (*n* = 1,748)	*χ* ^2^	*p*
Food allergen						
Egg	84 (28.28%)	124 (22.92%)	58 (9.34%)	39 (2.23%)	342.31	<0.001
Crab	24 (8.08%)	52 (9.61%)	60 (9.66%)	166 (9.50%)	0.702	0.873
Milk	84 (28.28%)	86 (15.90%)	32 (5.15%)	19 (1.09%)	374.898	<0.001
Beef	69 (23.23%)	57 (10.54%)	14 (2.25%)	20 (1.14%)	307.958	<0.001
Marine fish	22 (7.41%)	39 (7.21%)	35 (5.64%)	53 (3.03%)	24.785	<0.001
Mutton	49 (16.50%)	30 (5.55%)	26 (4.19%)	35 (2.00%)	129.956	<0.001
Shrimp	14 (4.71%)	31 (5.73%)	24 (3.86%)	28 (1.60%)	29.948	<0.001
Soybean	21 (7.07%)	26 (4.81%)	12 (1.93%)	35 (2.00%)	32.049	<0.001
Peanut	17 (5.72%)	22 (4.07%)	12 (1.93%)	25 (1.43%)	28.357	<0.001
Inhalant allergen						
Dust mite	55 (18.52%)	265 (48.98%)	337 (54.27%)	475 (27.17%)	229.337	<0.001
Cat hair	19 (6.40%)	28 (5.18%)	32 (5.15%)	84 (4.81%)	1.358	0.715
Cockroach	0 (0.00%)	15 (2.77%)	29 (4.67%)	119 (6.81%)	32.89	<0.001
House dust	2 (0.67%)	25 (4.62%)	60 (9.66%)	39 (2.23%)	76.448	<0.001
Tree	4 (1.35%)	20 (3.70%)	31 (4.99%)	81 (4.63%)	8.047	0.045
Mold	6 (2.02%)	16 (2.96%)	28 (4.51%)	31 (1.77%)	14.656	0.002
Mugwort	7 (2.36%)	9 (1.66%)	15 (2.42%)	81 (4.63%)	15.399	0.002
*Humulus scandens*	0 (0.00%)	1 (0.18%)	8 (1.29%)	22 (1.26%)	8.580	0.035
Ragweed	4 (1.35%)	17 (3.14%)	21 (3.38%)	76 (4.35%)	7.359	0.061
Dog epithelium	8 (2.69%)	11 (2.03%)	12 (1.93%)	26 (1.49%)	2.558	0.465

Data are presented as positive counts (*n*) and percentages based on the total testing records in each age group (*n* = 297, 541, 621, and 1,748, respectively). Denominators are testing records, not unique patients. *χ*^2^ and *p* values are from chi-square tests for comparisons across age groups.

### 3.5 Secondary analysis: analysis of allergen-specific IgE (sIgE) antibody status in patients with allergic diseases of different genders

**Figure 5:**
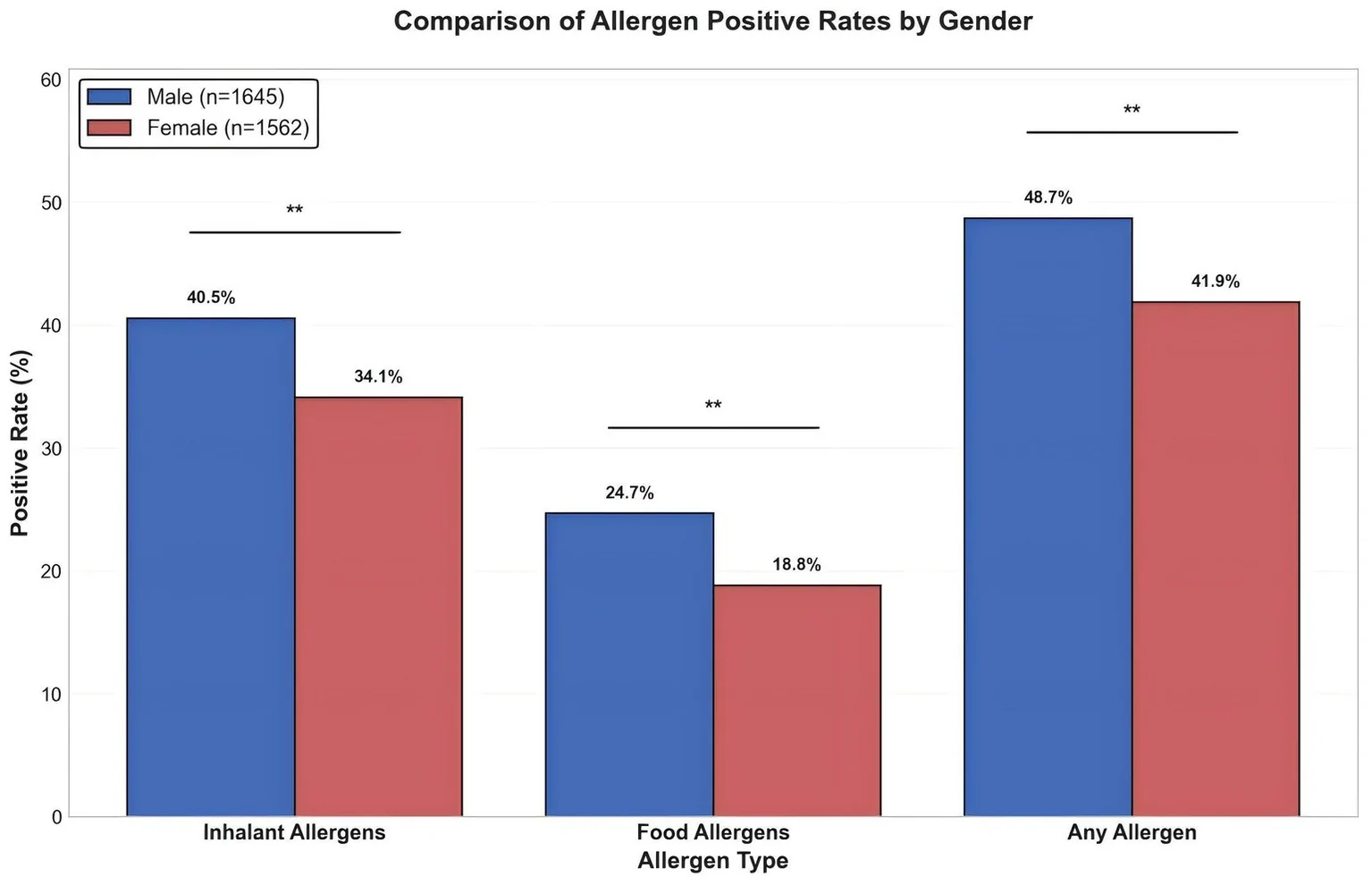
Grouped bar chart comparing allergen-specific IgE positive rates stratified by gender across three allergen categories: inhalant allergens, food allergens, and any allergen. Blue bars represent male patients (*n* = 1,645), red bars represent female patients (*n* = 1,562). The vertical axis indicates positive rate (%). Double asterisks (^**^) denote statistically significant intergender differences (*p* < 0.01) within each allergen category. Male patients exhibited significantly higher positive rates than females for all three allergen subgroups.

Figure 5 and Table 5 presents data on the positive rates of allergen-specific IgE (sIgE) between male and female patients. A notable finding is the significant difference in positive rates between male and female patients, with higher rates observed in males across both inhalant and food allergens. Specifically, males exhibited higher positive rates for inhalant allergen sIgE (*χ*^2^ = 14.121, *p* < 0.01) and food allergen sIgE (*χ*^2^ = 16.118, *p* < 0.01) compared to females. Furthermore, the positive rates of inhalant allergen sIgE were significantly higher than those of food allergen sIgE in both male (*χ*^2^ = 94.213, *p* < 0.01) and female (*χ*^2^ = 93.938, *p* < 0.01) patients. These findings shed light on gender differences in allergen sensitivity, emphasizing the need for tailored approaches to diagnosis and management based on patient gender.

**Table 5:** Positive rates of allergens in the inhalant and food groups by gender.

Allergens	Male (*n* = 1,645)		Female (*n* = 1,562)		*χ* ^2^	*p*
	Positive	Positive rate	Positive	Positive rate		
Inhalant allergen	667	40.55%	533	34.12%	14.121	<0.01
Food allergen	406	24.68%	294	18.82%	16.118	<0.01
Inhalant or food	801	48.69%	653	41.81%	15.337	<0.01

Percentages are based on total testing records for each gender group (male: *n* = 1,645; female: *n* = 1,562). Denominators are testing records, not unique patients.

## 4 Discussion

Our retrospective analysis of 3,207 testing records (including repeated episodes from some patients) from patients with suspected allergic diseases in Xiamen from 2019 to 2023 revealed that dust mite was the predominant allergen, with an overall positivity rate of 35.30%, followed by crab (9.42%), egg (9.51%), and milk (6.89%). Notably, the annual sIgE positivity rate showed a progressive decline from 76.07% in 2019 to 32.26% in 2023, with statistically significant differences between 2022/2023 and pre-pandemic 2019. Age-stratified analysis demonstrated that dust mites were more prevalent in children under 18, whereas adults showed higher rates of sensitization to cockroaches. Food allergen sensitization shifted from hen’s egg white and milk in infants to crab and seafood combinations in adults. Males exhibited significantly higher sIgE positivity rates for both inhalant and food allergens compared to females. These findings provide updated epidemiological evidence on allergen sensitization patterns in Xiamen, particularly against the backdrop of distinct lifestyle changes induced by the COVID-19 pandemic.

The consistent predominance of dust mites as the leading inhalant allergen in Xiamen warrants regional contextualization. Xiamen’s subtropical coastal climate, characterized by elevated annual temperatures (averaging 21 °C) and high relative humidity (mean ~80%) (15), creates an optimal microenvironment for the proliferation of dust mites, particularly *D. pteronyssinus* and *D. farinae*, in carpets, pillows, mattresses, and upholstered furniture. This climatological explanation aligns with observations from other humid coastal or riverine cities in China. For instance, a study from Ningbo, another coastal eastern Chinese city with a comparable humid subtropical monsoon climate, reported house dust mite sensitization as the leading allergen among 19,787 patients (37.0%) (16). Similarly, in Suzhou, dust mite mix was identified as the top inhalant allergen (17). These convergent findings underscore that high humidity and temperate conditions are critical drivers of dust mite allergen burden across regions sharing similar ecological characteristics (18, 19). Such environmental consistency reinforces the recommendation that diagnostic panels and prevention strategies in coastal southeastern China prioritize house dust mites as a primary target.

While the following mechanisms remain speculative and were not directly measured in this study, several hypotheses have been proposed in the literature to contextualize the observed trends. Nonetheless, the association between the pandemic period and declining sensitization rates should not be interpreted as causal. The observed downward trajectory contrasts with several domestic studies that reported unchanged or increased sensitization rates during the same period (20, 21). These divergent trends may reflect regional differences in testing practices, population composition, and NPI stringency. In Xiamen, sustained NPIs—including mandatory mask-wearing, home confinement, remote working, and reduced outdoor activities—may have been associated with reduced exposure to environmental allergens. Furthermore, improved household hygiene practices may have contributed to lower indoor allergen levels. However, whether NPIs contributed to this observed trend cannot be determined from the present data, as individual NPI adherence and actual allergen exposure levels were not directly measured. The precise interplay between NPIs, hygiene changes, and allergen sensitization trends therefore remains incompletely understood and requires further investigation. The application of Bonferroni correction for multiple pairwise comparisons (Supplementary Table S2) provided a conservative assessment of interannual differences. While the crude comparisons between 2019 and 2021 suggested a significant difference (*p* = 0.013), the corrected *p* value (adjusted *p* = 0.130) did not reach statistical significance, indicating that the 2021 decline was not robust enough to withstand correction for multiple testing. In contrast, the differences involving 2022 and 2023 remained highly significant even after the most stringent adjustment (all adjusted *p* < 0.001), reinforcing the conclusion that the decline became unequivocally established from 2022 onward.

Beyond these mechanistic speculations, an important methodological consideration merits attention. The most parsimonious explanation for the observed decline in crude annual positivity rates is the marked expansion of the tested population—from 117 testing records in 2019 to 1,609 in 2023—rather than a genuine biological decrease in sensitization. The 2019 sample was small and likely drawn from a more severely affected referral population, whereas the progressively larger samples in subsequent years included increasingly broader and milder cases, driving down the crude positivity rate through spectrum bias alone. This interpretation is supported by the fact that absolute sensitization counts for nearly all allergens increased in absolute terms over the study period over the same period (Table 3). Nevertheless, when we adjusted for age and sex using GEE, the sensitization risk in 2022 (aOR = 0.30, 95% CI: 0.19–0.48) and 2023 (aOR = 0.24, 95% CI: 0.15–0.37) remained significantly lower than that in 2019, indicating that while spectrum bias contributed to the declining trend, it does not fully account for it. Notably, the 2021 aOR (0.49) became statistically significant in the GEE analysis, whereas the unadjusted comparison was not significant after Bonferroni correction (adjusted *p* = 0.130). This discrepancy suggests that age and sex composition in 2021 partially masked the true decline, underscoring the importance of multivariable adjustment in this dataset.

The baseline characteristics of the study population also warrant attention. The marked fluctuations in age distribution (Supplementary Table S1)—with median age dropping to 11 years in 2021—underscore the heterogeneity in patient composition across years. This observation supports our decision to perform multivariable adjustment for age and sex in the primary analysis. Notably, the proportion of inpatient visits increased substantially from 0% in 2019 to 30.1% in 2022, reflecting changes in testing indications over time. While our multivariable model could not directly adjust for visit type due to collinearity with year, the inclusion of age and sex as covariates partially accounted for the underlying differences in case mix. A methodological strength of this study is the use of Generalized Estimating Equations (GEE) with robust standard errors to account for within-individual correlation arising from repeated sIgE tests in some patients. This approach ensures that the reported confidence intervals and *p* values are not overly optimistic due to clustering of observations, thereby providing more reliable estimates of the independent effects of year, age, and sex on sensitization. Future studies should consider prospective enrollment with standardized clinical assessments to further mitigate these potential biases.

Regarding food allergens, our finding that crab is a predominant food allergen in Xiamen among adults is noteworthy and extends the findings of a prior Xiamen study (22), which identified crab as the third most common food allergen (4.07%) in patients with skin allergic diseases, by demonstrating its sustained importance in a larger cohort and across a longer time series. While nationwide surveys have identified shrimp as the most common seafood allergen in China (23), our results highlight the local importance of crab—likely reflecting the prominent role of crab in Xiamen’s coastal culinary culture and high per-capita consumption. Age-specific analysis revealed a clear transition: infant food allergies are dominated by hen‘s egg white and milk, consistent with the early introduction of egg- and milk-based complementary foods and immature gastrointestinal barrier function, which facilitates the absorption of macromolecular antigens. In contrast, crab and marine fish combinations become the leading food allergens in adolescents and adults, suggesting that sensitization to seafood allergens accumulates with age through repeated environmental and dietary exposures (24). These age-dependent patterns, if validated with clinical symptom data, may inform the selection of allergens for diagnostic workups. However, as our data reflect sensitization rather than confirmed allergy, these observations should be considered hypothesis-generating for the optimization of diagnostic approaches in the region (25).

With respect to gender differences, we observed that males demonstrated significantly higher sIgE positivity rates for both inhalant and food allergens compared to females. While some studies have reported that females are more sensitive to certain allergens such as cat hair and dust mites, our findings align with a growing body of literature indicating that males, particularly before puberty, have a higher prevalence of IgE sensitization. A study from Ningbo similarly reported that male patients showed higher sensitization rates to multiple allergens than females. Immunologically, these differences may be attributable to distinct effects of sex hormones on immunoglobulin production and on the innate and adaptive immune systems: estrogens generally enhance humoral immune responses, whereas androgens exert suppressive effects on mast cell reactivity and Th2 cell differentiation (26).

Several limitations of this study should be acknowledged. First, as a retrospective cross-sectional analysis of hospital-based data from a single center, our results may not be fully generalizable to the general population of Xiamen or to other regions with different ecological and demographic characteristics. Second, the absence of a healthy control group precludes direct estimation of population-level sensitization prevalence. Third, the relatively small sample size in early pandemic years (e.g., *N* = 117 in 2019) reduces statistical stability for annual comparisons. Fourth, data on NPIs such as actual mask-wearing duration, household cleaning frequency, and allergic individuals’ COVID-19 infection status were not systematically collected, limiting causal inference for the observed pandemic-associated trends. Fifth, sIgE positivity indicates sensitization rather than clinically manifest allergy, and some positive results may reflect asymptomatic sensitization or cross-reactivity. Moreover, we lacked access to key clinical information, including oral food challenge test results, physician-confirmed allergy diagnoses, and longitudinal follow-up data for individual patients, which precluded further validation of sensitization findings against clinical outcomes. Seventh, a limitation of using this fixed panel is that it may not include all regionally relevant allergens (e.g., specific local pollen or fungal species not represented in the panel), potentially underestimating sensitization to certain locally important allergens. Future studies with larger sample sizes and prospective designs may further investigate whether the intensity of sensitization (as reflected by sIgE semi-quantitative grades) has also shifted over time.

## 5 Conclusion

This study, based on 3,207 serum sIgE testing records, delineates a comprehensive profile of allergen sensitization patterns in Xiamen between 2019 and 2023. Dust mites remain the predominant allergen, consistent with Xiamen’s subtropical coastal climate. Notably, the annual sIgE positivity rate exhibited a significant decline from 2021 onward, with the lowest rate observed in 2023, a trend that remained significant after adjustment for age, sex, and within-individual correlation, contrasting with findings from other regions. However, because we did not directly measure environmental allergen levels or individual adherence to non-pharmaceutical interventions (NPIs), this observed decline cannot be attributed to NPIs; as discussed in the preceding section, spectrum bias (e.g., changes in test-seeking behavior or referral patterns over the pandemic period) offers a more parsimonious explanation than a direct causal effect. Age-specific and gender-specific differences underscore the importance of tailored diagnostic approaches. These findings may inform the selection of allergens for diagnostic panels, pending validation with symptom data and provide epidemiological reference data for future hypothesis-driven studies when combined with symptom data. These work contributes updated regional evidence but the temporal patterns should be interpreted with caution; further multicenter prospective studies are warranted to validate these observations and to disentangle the relative contributions of behavioral changes, environmental factors, and diagnostic shifts that may underlie the observed sensitization trends.

## Data Availability

The original contributions presented in the study are included in the article/Supplementary material, further inquiries can be directed to the corresponding authors.

## References

[1] Ansotegui IJ Melioli G Canonica GW Caraballo L Villa E Ebisawa M . IgE allergy diagnostics and other relevant tests in allergy, a world allergy organization position paper. World Allergy Organ J. (2020) 13:100080. doi: 10.1016/j.waojou.2019.100080, 32128023 PMC7044795

[2] Skypala IJ Jeimy S Brucker H Nayak AP Decuyper II Bernstein JA . Cannabis-related allergies: an international overview and consensus recommendations. Allergy. (2022) 77:2038–52. doi: 10.1111/all.15237, 35102560 PMC9871863

[3] Gould HJ Sutton BJ. IgE in allergy and asthma today. Nat Rev Immunol. (2008) 8:205–17. doi: 10.1038/nri2273, 18301424

[4] Hao GL Xie TL Jing XY Kang S Liu TT Wang ZF . Analysis of allergen-specific IgE test results in children with allergic diseases in a women and children's hospital in Qingdao City. Zhonghua Yu Fang Yi Xue Za Zhi. (2026) 60:382–9. doi: 10.3760/cma.j.cn112150-20250815-00794 41856624

[5] Riggioni C Ricci C Moya B Wong D van Goor E Bartha I . Systematic review and meta-analyses on the accuracy of diagnostic tests for IgE-mediated food allergy. Allergy. (2024) 79:324–52. doi: 10.1111/all.15939, 38009299

[6] Riggioni C Oton T Carmona L Du Toit G Skypala I Santos AF. Immunotherapy and biologics in the management of IgE-mediated food allergy: systematic review and meta-analyses of efficacy and safety. Allergy. (2024) 79:2097–127. doi: 10.1111/all.16129 38747333

[7] Quintero-Campos P Gozalbo-Rovira R Rodríguez-Díaz J Maquieira Á Morais S. Standardizing in vitro β-lactam antibiotic allergy testing with synthetic IgE. Anal Chem. (2023) 95:12113–21. doi: 10.1021/acs.analchem.3c02284, 37545056 PMC10859892

[8] Martínez-Cañavate A Mesa-Del-Castillo M Carballada F Rivas-Juesas C Porto JÁ Blasco C . Molecular signatures of aeroallergen sensitization in pediatric populations: a comparative study across Spanish cities. Int J Mol Sci. (2025) 26:2963. doi: 10.3390/ijms26072963, 40243575 PMC11988508

[9] Khanolkar RA Trajkovski A Agarwal A Pauls MA Lang ES. Emerging evidence for non-pharmacologic interventions in reducing the burden of respiratory illnesses. Intern Emerg Med. (2022) 17:639–44. doi: 10.1007/s11739-022-02932-y, 35119570 PMC8814568

[10] Hurley S Franklin R McCallion N Byrne AM Fitzsimons J White M . Atopic outcomes at 2 years in the CORAL cohort, born in COVID-19 lockdown. Pediatr Allergy Immunol. (2023) 34:e14013. doi: 10.1111/pai.14013, 37747751

[11] Caid K Tate M Yousuf S Jones L Pesek RD Jefferson AA . Effects of nonpharmaceutical interventions during the COVID-19 pandemic on pediatric asthma exacerbations and viral infections. J Allergy Clin Immunol Glob. (2024) 3:100340. doi: 10.1016/j.jacig.2024.100340 PMC11533078 39498233

[12] Olbrich H Preuß SL Kridin K Hernandez G Thaçi D Ludwig RJ . COVID-19 infection raises respiratory type 2 inflammatory disease risk, whereas vaccination is protective. J Allergy Clin Immunol. (2026) 157:517–24. doi: 10.1016/j.jaci.2025.07.030, 40812431

[13] Zhang Y Fu X Chen Y Yang F Xing Z Li D . Divergent sensitization kinetics to indoor allergens in children: early dust mite surges vs delayed cat dander peaks during COVID-19 lockdowns. J Asthma Allergy. (2025) 18:1539–49. doi: 10.2147/JAA.S556457, 41216305 PMC12596836

[14] Qiu X Shi J Gu H Tian J. Shifting patterns of allergen sensitization in 8,797 children with atopic dermatitis during the COVID-19 restriction measures: a retrospective study from Hangzhou, China. Int Arch Allergy Immunol. (2026):1–10. doi: 10.1159/000551204, 41734138

[15] China Weather Network. Xiamen Travel: Climate. Beijing: China Weather Network (2026).

[16] Tu J Li J Xie Q Hu C Cheng P Wang Y. Hospital-based patterns of allergen sensitization among 19,787 patients with suspected allergic diseases in Ningbo, China. Front Public Health. (2026) 14:1786052. doi: 10.3389/fpubh.2026.1786052, 42110272 PMC13152857

[17] Wang WJ Du XM Ye L Wang XL Zhang GY. Distribution of serum specific IgE in children with allergic conjunctivitis and analysis of its concomitant allergic diseases. Transl Pediatr. (2020) 9:636–44. doi: 10.21037/tp-20-216, 33209726 PMC7658764

[18] Sun BQ Lai XX Gjesing B Spangfort MD Zhong N-S. Prevalence of sensitivity to cockroach allergens and IgE crossreactivity between cockroach and house dust mite allergens in Chinese patients with allergic rhinitis and asthma. Chin Med J. (2010) 123:3540–4. doi: 10.3760/cma.j.issn.0366-6999.2010.24.007 22166627

[19] Gan H Luo WT Huang ZF Zhang T Hou XQ Chen YW . House dust mite components sensitization profile in China, a multi-Centre study. Clin Exp Allergy. (2023) 53:226–9. doi: 10.1111/cea.14255, 36519672

[20] Li Y Hu H Zhang T Wang G Huang H Zheng P . Increase in indoor inhalant allergen sensitivity during the COVID-19 pandemic in South China: a cross-sectional study from 2017 to 2020. J Asthma Allergy. (2017) 14:1185–95. doi: 10.2147/JAA.S322034, 34616158 PMC8488032

[21] Wang PL Xu LN Jiang WJ Li PY Ma Y Dai YF . A study of allergen-specific IgE in children in Suzhou from 2018 to 2021. J Clin Pediatr. (2024) 42:198–203.

[22] Lin YR Yu Y Ouyang L Zhang L Song WF. Analysis of allergen detection results in 2967 patients with skin allergic diseases in Xiamen. Int J Lab Med. (2022) 43:2781–2785, 2792. doi: 10.3969/j.issn.1673-4130.2022.22.019

[23] Huang Z Feng W Wei W Yang B Wang L. Prevalence of food-allergen and aeroallergen sensitization among people in Sichuan, Western China: an 8-year observational study. J Clin Lab Anal. (2019) 33:e22723. doi: 10.1002/jcla.22723, 30461057 PMC6818623

[24] Liu MT Liu L Qi WT Zheng XH Chen JX Yao JN . Interpreting epidemiologic distribution of total and specific IgE levels for food allergy in southern China from 2004 to 2023: understanding the mechanisms and focusing on prevention. BMC Public Health. (2024) 24:3022. doi: 10.1186/s12889-024-20470-4, 39482634 PMC11529331

[25] Leung AS Jie S Gu Y Wong GW. Food allergy in children in China. Clin Exp Allergy. (2025) 55:634–47. doi: 10.1111/cea.14596, 39641430 PMC12325142

[26] Leffler J Stumbles PA Strickland DH. Immunological processes driving IgE sensitization and disease development in males and females. Int J Mol Sci. (2018) 19:1554. doi: 10.3390/ijms19061554 PMC6032271 29882879

