# Supplementary material for: Temporal changes in allergen-specific IgE sensitization patterns in Xiamen, 2019–2023: a retrospective cross-sectional analysis
Source: Front Public Health. 2026 Sep 11;14:1916258. doi: 10.3389/fpubh.2026.1916258 (PMC13612525; doi:10.3389/fpubh.2026.1916258)
Supplement: Supplementary file 1 [file Table_1.pdf]

Supplementary Table S1. Baseline characteristics of the study testing records from patients with suspected allergic diseases in Xiamen, 2019–2023.

Year	N	Age	Gender, n(%)		Visit type, n(%)	
		median (P25, P75)	Male	Female	Outpatient	Inpatient
2019	117	21(4, 32)	63(53.8)	54(46.2)	117(100.0)	0(0.0)
2020	273	20(5, 31)	130(47.6)	143(52.4)	241(88.3)	32(11.7)
2021	377	11(4, 34)	187(49.6)	190(50.4)	311(82.5)	66(17.5)
2022	831	22(5, 38)	455(54.8)	376(45.2)	581(69.9)	250(30.1)
2023	1609	22(6, 36)	810(50.3)	799(49.7)	1288(80.1)	321(19.9)

Data are based on 3,207 testing records. Denominators represent testing records, not unique patients. Age is presented as median (P25, P75). Data are presented as n (%) for categorical variables. Percentages may not sum to 100% due to rounding.

Supplementary Table S2. Pairwise comparisons of annual sIgE positivity rates from 2019 to 2023 (χ^2 test) based on testing records.

Comparison	χ^2	Raw P	Adjusted P value*	Significance
2019vs2020	2.065	0.151	1.000	ns
2019vs2021	6.180	0.013	0.130	ns
2019vs2022	33.760	<0.001	<0.001	***
2019vs2023	91.755	<0.001	<0.001	***
2020vs2021	1.907	0.167	1.000	ns
2020vs2022	28.599	<0.001	<0.001	***
2020vs2023	133.366	<0.001	<0.001	***
2021vs2022	18.644	<0.001	<0.001	***
2021vs2023	127.577	<0.001	<0.001	***
2022vs2023	75.431	<0.001	<0.001	***

Pairwise comparisons were performed using Pearson's chi-square test. All P values are two-tailed. Adjusted P values were calculated using the Bonferroni method for 10 comparisons (adjusted P = raw P × 10, capped at 1.00). Significance was defined as adjusted P < 0.05. ns: adjusted P ≥ 0.05 (not significant); *: adjusted P < 0.001.**

Supplementary Table S3. Full multivariable analysis results for overall sensitization (any sIgE positive) using Generalized Estimating Equations (GEE) based on 2,784 testing records clustered by 2,750 unique patients.

Variable	B	SE	Wald χ²	df	P	aOR*	95% CI
Age group (vs <3 years)			124.372	3	<0.001		
3–5 years	0.474	0.162	8.559	1	0.003	1.61	1.17–2.21
>5–18 years	0.484	0.158	9.326	1	0.002	1.62	1.19–2.21
>18 years	–0.485	0.138	12.288	1	<0.001	0.62	0.47–0.81
Gender (vs female)			10.149	1	0.001		
Male	0.255	0.08	10.149	1	0.001	1.29	1.10–1.51
Year (vs 2019)			99.506	4	<0.001		
2020	–0.389	0.258	2.27	1	0.132	0.68	0.41–1.12
2021	–0.709	0.247	8.271	1	0.004	0.49	0.30–0.80
2022	–1.197	0.232	26.594	1	<0.001	0.30	0.19–0.48
2023	–1.447	0.230	39.73	1	<0.001	0.24	0.15–0.37
Constant	1.181	0.254	21.696	1	<0.001	3.26	1.98–5.36

aOR: adjusted odds ratio; CI: confidence interval. Adjusted for age group (<3, 3–5, >5–18, >18 years) and gender (female as reference) using Generalized Estimating Equations (GEE) with exchangeable correlation structure and robust standard errors. Year was entered as a categorical variable with 2019 as the reference; test for linear trend across years was performed by treating year as a continuous variable (P for trend < 0.001).
